# Slimmer or Fertile? Pharmacological Mechanisms Involved in Reduced Sperm Quality and Fertility in Rats Exposed to the Anorexigen Sibutramine

**DOI:** 10.1371/journal.pone.0066091

**Published:** 2013-06-12

**Authors:** Cibele S. Borges, Gabriela Missassi, Enio S. A. Pacini, Luiz Ricardo A. Kiguti, Marciana Sanabria, Raquel F. Silva, Thais P. Banzato, Juliana E. Perobelli, André S. Pupo, Wilma G. Kempinas

**Affiliations:** 1 Department of Morphology, Institute of Biosciences, UNESP - Univ Estadual Paulista, Botucatu, São Paulo, Brazil; 2 Department of Pharmacology, Institute of Biosciences, UNESP - Univ Estadual Paulista, Botucatu, São Paulo, Brazil; University of Iowa, United States of America

## Abstract

Sperm acquire motility and fertility capacity during epididymal transit, under the control of androgens and sympathetic innervations. It is already known that the acceleration of epididymal sperm transit time can lead to lower sperm quality. In a previous work we showed that rats exposed to the anorexigen sibutramine, a non-selective serotonin-norepinephrine reuptake inhibitor, presented faster sperm transit time, lower epididymal sperm reserves and potentiation of the tension of epididymal duct to norepinephrine exposed acutely *in vitro* to sibutramine. In the present work we aimed to further investigate pharmacological mechanisms involved in these alterations and the impact on rat sperm quality. For this, adult male *Wistar* rats were treated with sibutramine (10 mg/kg/day) or vehicle for 30 days. Sibutramine decreased final body, seminal vesicle, ventral prostate and epididymal weights, as well as sperm transit time in the epididymal cauda. On the contrary of the *in vitro* pharmacological assays, in which sibutramine was added directly to the bath containing strips of distal epididymal cauda, the ductal tension was not altered after *in vivo* sub-chronic exposure to sibutramine. However, there is pharmacological evidence that the endogenous epididymal norepinephrine reserves were reduced in these animals. It was also shown that the decrease in prostate weight can be related to increased tension developed of the gland, due to sibutramine sympathomimetic effects. In addition, our results showed reduced sperm quality after *in utero* artificial insemination, a more sensitive procedure to assess fertility in rodents. The epididymal norepinephrine depletion exerted by sibutramine, associated with decreases in sperm transit time, quantity and quality, leading to reduced fertility in this experimental model, reinforces the concerns about the possible impact on fertility of man taking sibutramine as well as other non-selective serotonin-norepinephrine reuptake inhibitors, especially considering the lower reproductive efficiency of humans compared to males of other species.

## Introduction

The epididymis is an organ of the male reproductive tract formed by highly convoluted duct that connects the efferent ducts to the vas deferens and performs a variety of functions, including sperm transport, maturation, protection, concentration and storage [1]. This organ plays an important role in the acquisition of progressive sperm motility and fertilizing ability. Alterations on the sperm transit time through the epididymis may compromise the sperm maturation process, which is modulated by androgens and contractile activity of epididymal smooth muscle layers [2], [3], [4]. The rat epididymis receives sympathetic and parasympathetic autonomic innervations from hypogastric and pelvic nerves, respectively [5], [6]. The contractions of the epididymal cauda induced by norepinephrine (NE) released by sympathetic nerve stimulation occur via activation of α_1_- adrenoceptor (α_1_-AR) [7].

Another organ that receives sympathetic and parasympathetic autonomic innervation is the prostate. This accessory organ of the male reproductive tract has the function to produce an alkaline secretion that composes the seminal fluid, improving sperm fertility potential and motility. The sympathetic stimulation (noradrenergic), via activation of α_1_-AR, contracts the prostatic smooth muscle cells releasing prostatic secretions into the urethra [8].

Since the sympathetic innervation is necessary for the structural and functional integrity of the epididymis and prostate, the exposure to drugs that acts on the nervous system, with known effects on the peripheral nervous system, could impair the morpho-physiology of these reproductive organs [9], [10], [11], [12].

Sibutramine, an appetite suppressant administered orally for obesity treatment [13], acts on the central nervous system as a non-selective serotonin-NE reuptake inhibitor, with subsequent activation of adrenoceptors, especially α_1_-ARs [14], [15], [16]. The anorexigenic activity of sibutramine promotes increase in energy expenditure and induction of peripheral sympathomimetic effects [17], [18], such as on the cardiovascular system [19] and on male reproduction [20]. Abnormal ejaculation was observed in rats, as well as an increased sensitivity to NE induced by the activation of the α_1_-ARs in tissues such as vas deferens and seminal vesicles, resulting in an increased tension developed [12]. In addition, sibutramine promoted an acceleration of sperm transit time in epididymal cauda due to its sympathomimetic effects [20].

Therefore, the aim of the present work was to further evaluate sibutramine effects, a serotonin-NE reuptake inhibitor, on the contractility of the epididymis and prostate, and its impact on sperm quality and fertility of adult male rats.

## Materials and Methods

### Ethics Statement

The experimental procedures were approved by the local Ethics Committee for the Use of Experimental Animals of the University of São Paulo State (protocol number 248-CEEA) and are in accordance with the Guide for the Care and Use of Laboratory Animals (National Institutes of Health). All surgery process was performed under ketamine-xylazine or urethane anesthesia and the euthanasia was performed by decapitation, all efforts were made to minimize suffering.

### Animals

Male (110 days old/490–540 g) and female (70 days old/220–270 g) *Wistar* rats were obtained from the Central Biotherium of UNESP – Univ Estadual Paulista, and maintained under controlled conditions (25°C, 30% air humidity, 12/12-h light/dark cycle) with food and water available *ad libitum*.

The male rats were randomly allocated into two groups: control, consisted of rats that were treated by gavage with the vehicle solution (33.3% dimethylsulfoxide and 66.7% saline) in 0.5 ml/kg, and treated, consisted of rats that were administered with sibutramine (10 mg/kg/day) in vehicle solution by gavage for 30 days [20].

### Drugs and Solutions

Drugs were obtained from the following sources: sibutramine hydrochloride monohydrate from Deg (Jiangyin Eas, China); cocaine from Boehringer (Phoenixville, USA); prazosin from Research Biochemicals (Massachusetts, USA), methoxamine hydrochloride, nifedipine, tyramine, and norepinephrine ((L)-(−)-NE bitartrate salt monohydrate) and dimethylsulphoxide (DMSO) from Sigma (St. Louis, MO, USA).

Sibutramine was diluted in 33.3% dimethylsulfoxide and 66.7% saline and administered once a day in 0.5 ml/kg. This dose was chosen as the minimum anorectic dose in this experimental model. The study was conducted in two steps, Experiment 1 and Experiment 2, and described as follows.

### Experiment 1: Reproductive Organ Weights, Serum Hormone Levels, Sperm Parameters, Fertility Assessment

Adult male rats (n = 8) were treated with 10 mg/kg/day of sibutramine as described previously. The respective control animals (n = 8) received only vehicle. Additionally, 10 male rats (110 days old) and 28 female (70 days old) were used in the fertility procedures.

#### Body and reproductive organs weights

The animals were weighed and euthanized by decapitation on the day after the end of treatment. The right testis, epididymis, vas deferens, ventral prostate, and seminal vesicle (without the coagulating gland) were removed and their weights recorded.

#### Hormonal measurements

After decapitation, blood was collected (between 9∶00 and 11∶30 AM) and serum was obtained by centrifugation (1236×*g*, for 20 min at 4°C). The concentrations of testosterone, luteinizing hormone (LH) and follicle-stimulating hormone (FSH) were determined by the technique of double antibody radioimmunoassay. Testosterone assay was performed using a TESTOSTERONE MAIA® kit (Biochem Immuno System). The LH and FSH assays were done using specific kits supplied by the National Institute of Arthritis, Diabetes and Kidney Diseases (NIADDK, USA). All samples were assayed in duplicate and in the same assay to avoid inter-assay errors. The intra-assay error was 3.4% for LH, 2.8% for FSH and 4% for testosterone.

#### Sperm counts, daily sperm production, and sperm transit time through the epididymis

Homogenization-resistant testicular spermatids (stage 19 of spermiogenesis) in the testis were counted as described previously [21], with adaptations adopted by Fernandes et al. [3]. Briefly, the testis, decapsulated and weighed soon after collection, was homogenized in 5 ml of NaCl 0.9% containing Triton X 100 0.5%, followed by sonication for 30 s. After a 10-fold dilution, one sample was transferred to Neubauer chambers (4 fields per animal), and mature spermatids were counted. To calculate the daily sperm production (DSP), the number of spermatids at stage 19 was divided by 6.1, which is the number of days of the seminiferous cycle during which these spermatids are present in the seminiferous epithelium. In the same manner, caput/corpus and cauda epididymidis were cut into small fragments with scissors and homogenized, and sperm counted as described for the testis. The sperm transit time through the epididymis was determined by dividing the number of sperm in each portion by DSP.

#### Fertility assessment

For this, *in utero* artificial insemination was used [22], [23], [24], [25], [26]. In brief, females in LHRH-induced proestrus were paired with sexually experienced, vasectomized males for 1 hour. Receptive females were selected for the insemination procedure. Eight male rats per group were used for sperm isolation as described elsewhere. Briefly, the sperm were released from the proximal epididymal cauda, first site where fertile sperm is encountered in the rat, by nicking the duct and collecting the sperm in 2 mL of modified human tubular fluid (HTF) medium (Irvine Scientific). After a 10-fold dilution, sperm were counted and each uterine horn was injected with a volume containing 5×10^6^ sperm [27]. One female was inseminated per male and when insemination was completed, the abdominal musculature was sutured. All surgery was performed under ketamine-xylazine anesthesia, and all efforts were made to minimize suffering.

Twenty days later, the females were euthanized by decapitation to enable fertility evaluation. After collection of the uterus and ovaries the numbers of corpora lutea, implants and reabsorptions were recorded and the following endpoints determined: fertility potential (efficiency of implantation): implantation sites/corpora lutea×100; rate of pre-implantation loss: (number of corpora lutea−number of implantations/number of corpora lutea)×100; rate of post-implantation loss: (number of implantations – number of live fetuses)/number of implantations×100.

#### Sperm motility

Sperm motility was evaluated in the same sperm sample used for artificial insemination. For this, an aliquot of 10 µL of sperm suspensions was immediately transferred to a Makler chamber maintained at 34°C. Using a phase-contrast microscope (400×magnification), 100 sperm were counted and classified as Type A (mobile with progressive movement) Type B (mobile without progressive movement) and Type C (immobile).

#### Sperm morphology

An aliquot of 100 µl from the same sample used for artificial insemination was added to 900 µl of formol saline. To analyze sperm morphologically, smears were prepared on histological slides that were left to dry for 90 min and 200 spermatozoa per animal were analyzed in a phase-contrast microscope (400×magnification). Morphological abnormalities were classified into two general categories: head morphology (without curvature, without characteristic curvature, pin head or isolated form, i.e., no tail attached) and tail morphology (broken or rolled into a spiral) [28]. Sperm were also classified as to the presence or absence of the cytoplasmic droplet.

### Experiment 2: Pharmacological Reactivity of the Epididymal Duct and Prostate

In this experiment, 37 adult male Wistar rats (110 days old) were used. Twenty-two rats were allocated into two experimental groups: Control and Sibutramine, following the same experimental design described in Experiment 1 and used for the evaluation of *in vitro* distal cauda epididymis duct tension after *in vivo* treatments.

Seven non-treated male rats were used for the evaluation of the effect of *in vitro* sibutramine administration on the tension of epididymal duct and 8 non-treated male rats were used for the ventral prostate tension *in vivo*.

The potency of NE, methoxamine and tyramine in inducing a tension in the smooth muscles cells of epididymal duct were expressed as pEC50 (used for *in vitro* experiment). This value of pEC50 is –log of NE, methoxamine or tyramine concentration inducing 50% of maximal smooth muscles cells tension. In the ventral prostate, the potency of methoxamine and NE in inducing a tension in the smooth muscles cells was expressed as pED50 (used for *in vivo* experiment). Because this value is –log of NE or methoxamine concentration inducing 50% of effective maximal smooth muscles cells tension.

#### Distal epididymal cauda isolation

The animals were killed by decapitation, the whole epididymis was carefully excised and isolated duct segments (1.5 cm) from the distal epididymal cauda were dissected to record of isometric tension as previously described [20]. After a 30 minutes stabilization period, the tissues were repeatedly challenged with 80 mM KCl to evaluate tissue viability and maximal response stabilization.

#### Effects of *in vitro* sibutramine administration on the tension developed of distal epididymal cauda

After mounting and stabilization of the tissues, a concentration-response curve to NE was obtained by the cumulative addition of the agonist (10^−9 ^M–10^−3 ^M) to the organ bath and this curve was taken as control curve. New concentration-response curves to NE were obtained in the presence of sibutramine (1, 3, and 7 µM) incubated for at least 30 minutes with the tissues and the maximal tension developed (Emax, in millinewtons - mN) and the potency of NE in inducing tension in the smooth muscles cells of epididymal duct (expressed as pEC_50_, the –log of NE concentration inducing 50% of maximal tension) were evaluated.

The dependence of the effects of sibutramine on the epididymal cauda tension on the neuronal NE reuptake system was investigated. To this end, the effect of sibutramine (3 µM) on the concentration-response curves to methoxamine (10^−7 ^M–10^−4 ^M), a α_1_-ARs-selective agonist that is a poor substrate for the neuronal NE reuptake system [29], was investigated. Tension in the presence of sibutramine was normalized to the maximal tension of control curves. In addition, as sibutramine *per se* induced tension of cauda epididymal duct, and this tension developed during 5 minutes by sibutramine (the sum of any phasic or tonic activity - total area produced in grafic) were recorded in the presence of nifedipine (300 nM) to investigate the dependence on the extracellular calcium influx through L-type voltage-dependent calcium channels.

#### *Ex vivo* assay: *In vitro* tension of the isolated distal epididymal cauda of animals treated with sibutramine or vehicle *in vivo*

The animals were weighed and euthanized by decapitation 24 hours after the last sibutramine administration. Strips from distal epididymal cauda from 8 animals/group were prepared for the *in vitro* tension recording as described above. After the stabilization period and challenge with 80 mM KCl a cumulative concentration–response curve to NE (10^−9^M–10^−3^M) was obtained and the maximal tension (Emax) and the potency of NE in developing tension of epididymal duct (pEC_50_) were evaluated. Another new concentration-response curve to NE was constructed in the presence of 6 µM cocaine to evaluate the functioning of neuronal NE reuptake system. Cocaine is a potent norepinephrine and dopamine reuptake inhibitor, prolonging the presence of these neurotransmitters in the synaptic cleft [30].

In addition, strips of epididymal cauda from 3 rats/group were isolated and mounted as described above. Concentration-response curves to tyramine (10^−7^M–10^−3^M), an amine that releases intravesicular NE from the sympathetic nerve terminals [31], [32] were evaluated. To ascertain whether the endogenous NE pool in the cauda epididymis was different between vehicle and sibutramine-treated rats the magnitude of antagonism of the α_1_-AR competitive antagonist prazosin (10 nM) against tyramine-induced tension was evaluated.

#### *In vivo* tension of ventral prostate

The *in vivo* prostate tension was evaluated as described by Kontani & Shiraoya [33]. Adult male rats (120 days old, 500 g weight) were anesthetized with urethane (1.5 g/kg, sc) and the prostate ventral lobes were tied with a cotton thread and attached to an isometric force transducer under 9.8 mN resting tension. The right jugular vein was catheterized with poliethylene tubing (PE10 connected to PE50) to intravenous drug administration and the body temperature was maintained by a heating lamp. After a 30 minutes stabilization period a cumulative dose-response curve to NE (10^−7^–10^−5^ g/kg) was constructed by intravenous administration of NE and this curve was considered as a control curve. Preliminary experiments showed that at least three consecutive concentration–response curves with phenylephrine (10^−6^–10^−4 ^g/kg) are similar in respect to sensitivity and maximal response (Figure S1). A new dose-response curve to NE was repeated 30 minutes after the control curve in the presence of sibutramine 5 mg/kg intravenously administrated 15 minutes before the curve construction. In another experiment, after a 30 minutes stabilization period a cumulative dose-response curve to methoxamine (10^−7^–10^−3^ g/kg) was constructed by intravenous administration of methoxamine and this curve was considered as a control curve and new dose-response curve to methoxamine was repeated 30 minutes after the control curve in the presence of sibutramine 5 mg/kg intravenously administrated 15 minutes before the curve construction. The tension developed (mN) was evaluated. All efforts were made to minimize suffering and in the end the animals were euthanized by decapitation.

### Statistical Analysis

Data are presented as mean ± standard error of mean (SEM) or median and interquartil range. Student’s t-test or ANOVA followed by Dunnet were used for comparison of parametric variables. Nonparametric variables were compared by Mann-Whitney test or Kruskal-Wallis followed by Dunn test. Spearman “r” coefficient was calculated to investigate possible correlations between fertility potential and sperm motility. Differences were considered significant when p<0.05. The statistical analyses were performed by GraphPad InStat (version 5).

## Results

### Experiment 1

**Figure 1:**
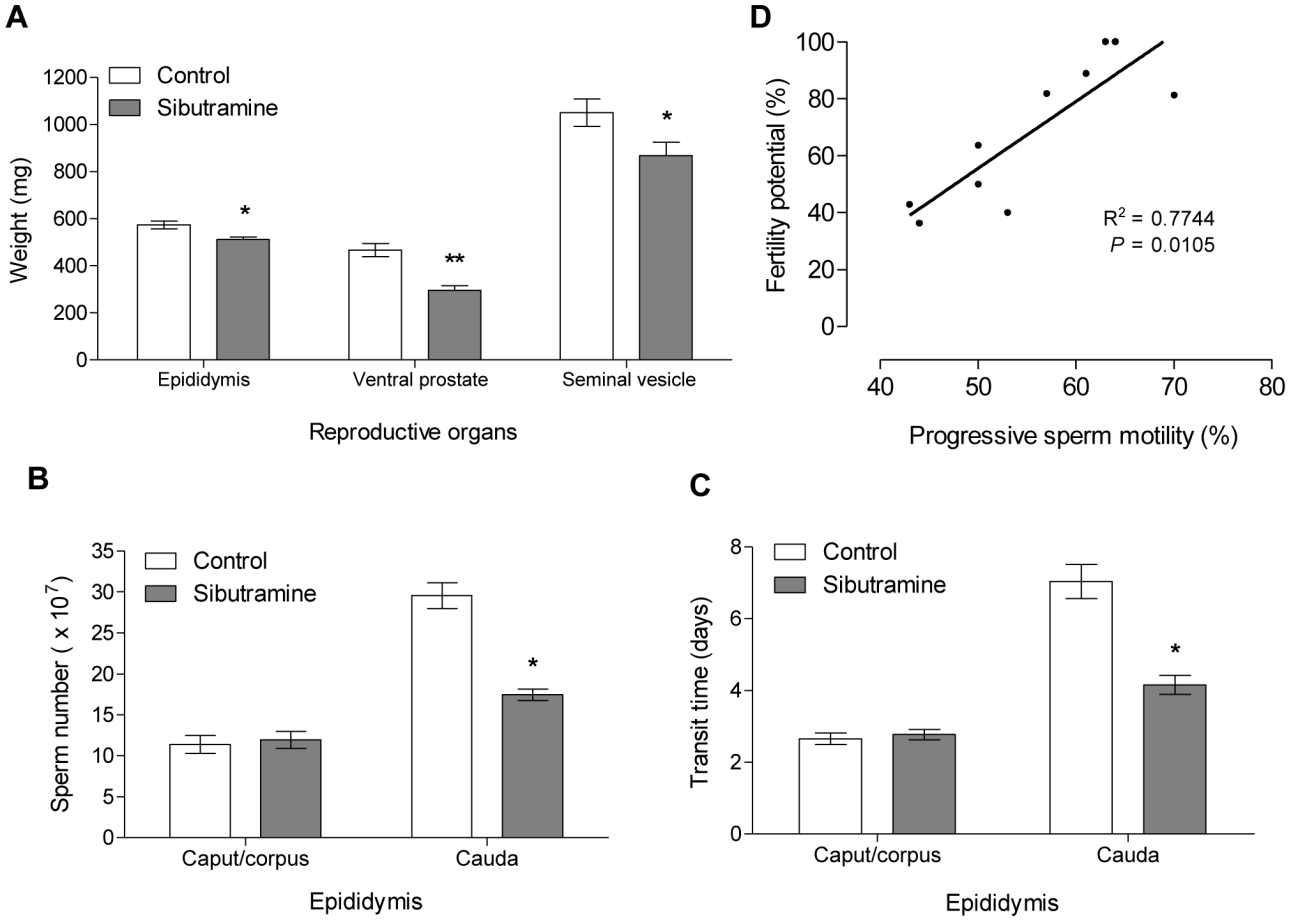
Reduction on reproductive organs weight, sperm reserves, sperm transit time and positive correlation between progressive sperm motility and fertility potential. **A:** Reproductive organs weight of the control and sibutramine-treated groups. **B:** Sperm reserves in caput/corpus and cauda of the control and sibutramine-treated groups. **C**: Sperm transit time in caput/corpus and cauda of the control and sibutramine-treated groups. **A–C:** The data are expressed as the mean±SEM. *p<0.05, **p<0,01 (Student's t- test). **D:** Scatter plot depicting the correlation between fertility potential (%) and progressive sperm motility (%); n = 10 (5 animals of the control group and 5 animals of the sibutramine-treated group). The linear equation for the graph was y = 2.355× –62.22, where y is equal to fertility potential and x corresponds to progressive sperm percentage.

At the end of the experiment, significant reduction in the body weight gain was observed in the sibutramine-treated group (−15.91±5.95 g) when compared with control group (18.38±3.88 g), as well as reductions in the weights of the epididymis, ventral prostate and seminal vesicle (Figure 1A).

There were no significant differences in serum levels of testosterone, LH, or FSH (Table S1), nor in the percentages of morphologically normal sperm between groups (96% control and 95% sibutramine group) and in the presence of cytoplasmic droplet (33% control and 48.5% sibutramine group). Sibutramine exposure did not alter sperm daily production, on the other hand, lead to a significant reduction in sperm reserves (Figure 1B) and transit time in the epididymal cauda (Figure 1C).

Fertility and sperm quality of proximal epididymal cauda sperm, assessed by *in utero* insemination, was significantly reduced by sibutramine exposure as shown by the significant reductions in fertility potential and pre-implantation loss rate (Table 1). There were no significant differences on sperm motility, but sperm with progressive motility and fertility potential were positively correlated (p<0.01) (Figure 1D).

**Table 1:** Fertility assessment after *in utero* artificial insemination.

Parameters	Control (n = 7)	Sibutramine (n = 7)
Pregnancy rate (%)	72	72
1Fertility potential (%)	88.89 (65.91–100.00)	40.00 (22.72–72.44)*
1Pre-implantation loss (%)	11.11 (0.00–34.09)	60.00 (24.84–77.27)*
1Post-implantation loss (%)	8.33 (0.00–68.75)	15.38 (7.00–62.50)
2Body weight of dams (g)	320.32±23.88	338.38±22.20
2Uterus weight with fetuses (g)	37.82±11.73	24.88±8.40
2Corpora lutea number	9.85±1.59	11.60±0.87
2Implantation number	8.81±1.93	5.80±2.03
2Number of live fetuses	7.60±2.31	4.80±1.82
2Fetus weight (g)	2.57±0.53	2.56±0.53

1 Values expressed as median and interquartile intervals *p<0.05 (Mann- Whitney test).

2 Values expressed as mean±SEM. (Student's t- test).

### Experiment 2

**Figure 2:**
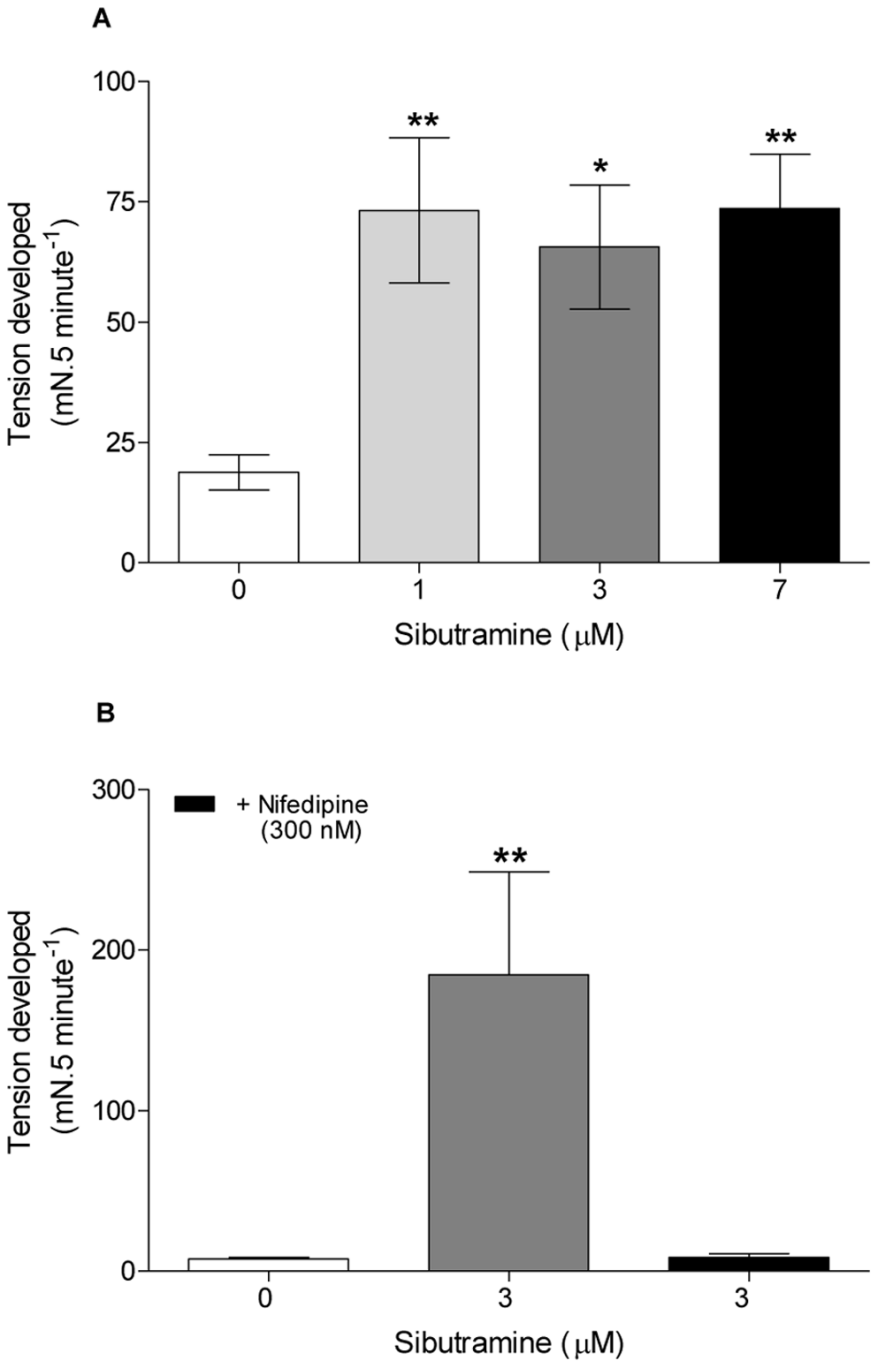
Effects of *in vitro* sibutramine on basal tonus activity of distal epididymal duct. **A**: Tension developed in 5 min in the absence and presence of increasing concentrations of sibutramine. The data are expressed as the mean±SEM. *p<0.05, **p<0.01 vs control (ANOVA followed by Dunnett test). **B:** Tension developed in 5 min in the presence of sibutramine 3 µM and nifedipine 300 nM. Data are expressed as the mean±SEM. **p<0.01 vs control (Student's t- test).

The basal tension (tension developed sum in 5 minutes) by the distal cauda epididymis duct increased (p<0.05) in the presence of different *in vitro* sibutramine concentrations (1, 3 and 7 µM) (Figure 2A). This tension was abolished in the presence of nifedipine (300 nM), a high affinity L-type calcium channel blocker (Figure 2B). This tension developed during 5 minutes is calculated as area under curve (Figure S2).

**Figure 3:**
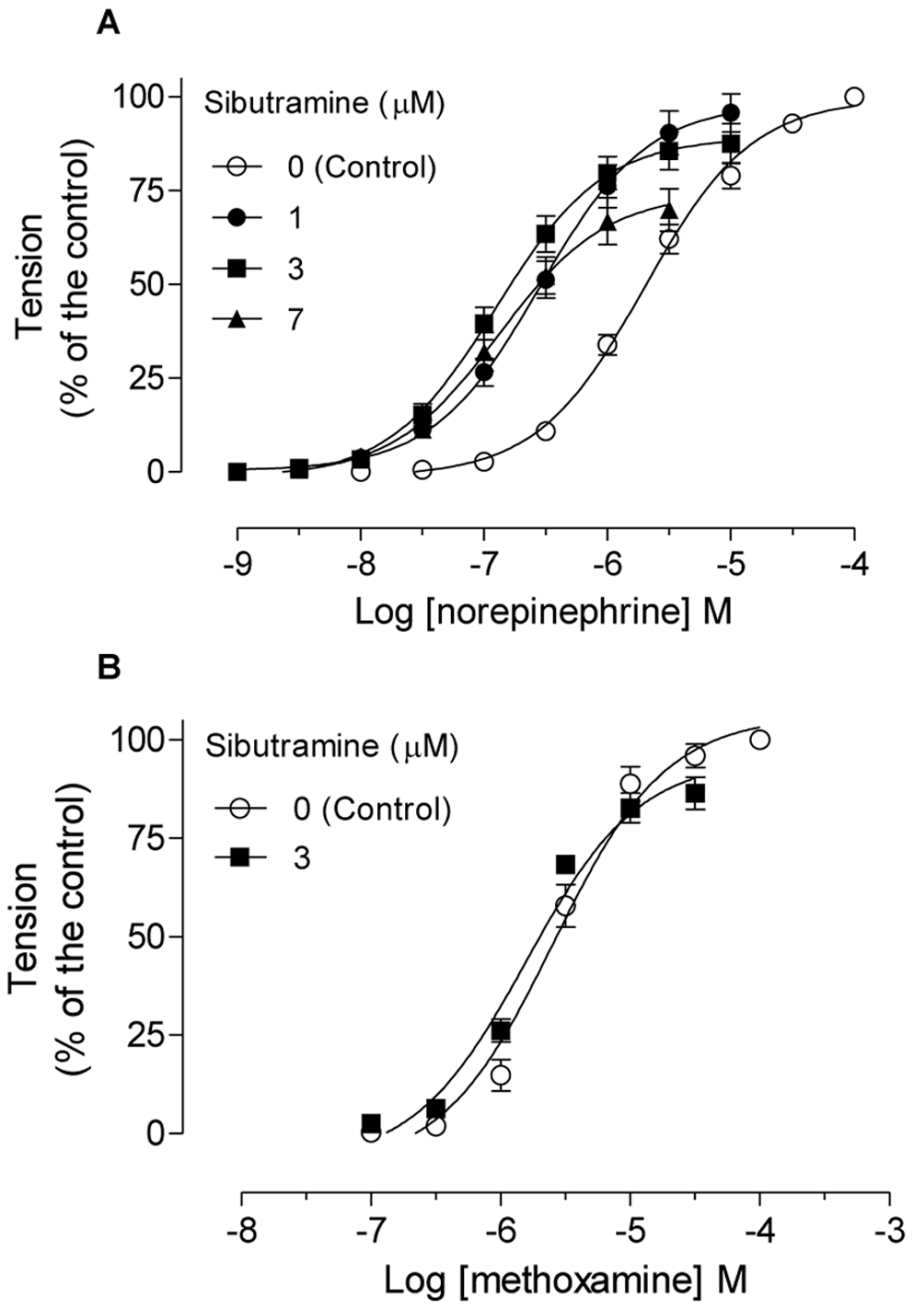
*In vitro* sibutramine administration increases the potency of NE-induced tension of epididymal cauda duct. **A:** Concentration-response curves to NE in the absence and presence of increasing concentrations of sibutramine in the epididymal cauda. **B:** Concentration-response curves to methoxamine in the absence and presence of sibutramine 3 µM in the epididymal cauda. **A–B:** The data are expressed as the mean±SEM. Values of pEC_50_ were different between NE curves, but not methoxamine curves (ANOVA, followed by Dunnett).

In the absence of sibutramine, NE induced concentration-dependent tension of the distal epididymal cauda with a potency of pEC_50_ = 5.697±0.041. Incubation with sibutramine 1, 3, and 7 µM increased the potency of the distal epididymal cauda to NE by approximately 8- (pEC_50_ = 6.580±0.069), 15 (pEC_50_ = 6.862±0.069), and 16-fold (pEC_50_ = 6.898±0.091), respectively. This increased potency (p<0.05) is shown by a leftward shift in the concentration-response curve to NE (Figure 3A). In addition, sibutramine 7 µM reduced the maximal tension developed by NE by approximately 29% (p<0.001).

To examine whether the increase in NE-developed tension of epididymal duct by sibutramine *in vitro* would be ascribed to the blocking of neuronal NE reuptake system, the effects of 3 µM sibutramine (a maximally effective sibutramine concentration in NE-induced tension) were investigated in tension of epididymal duct induced by the α_1_-ARs agonist methoxamine, a selective agonist which is a poor substrate for the reuptake system. No changes were observed on the maximal tension developed or in the potency of methoxamine in the epididymal duct (pEC_50_ control curve = 5.590±0.071; pEC_50_ sibutramine 3 µM = 5.802±0.086, p>0.05) (Figure 3B).

**Figure 4:**
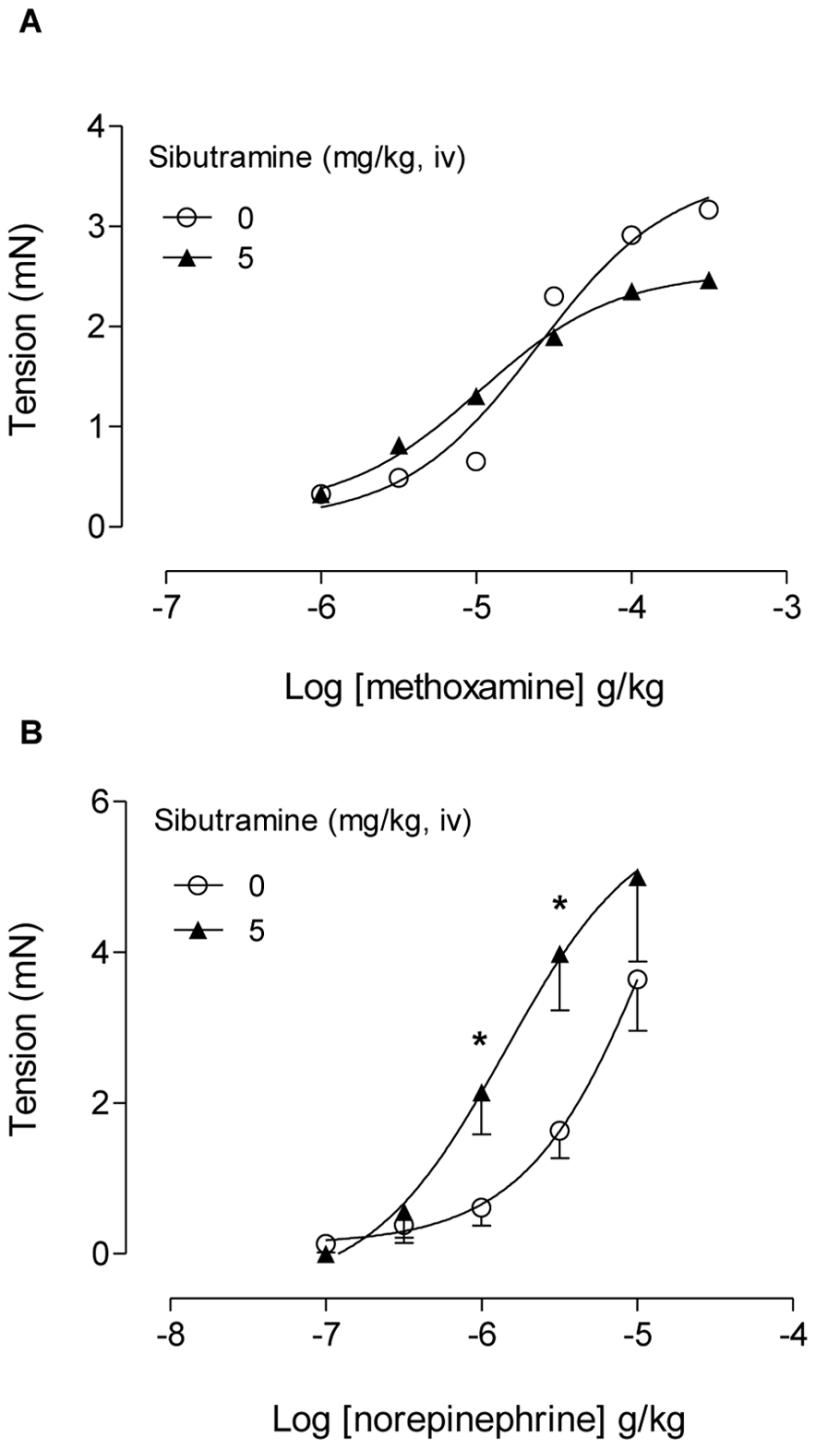
*In vivo* prostate tension to methoxamine and the tension increased to NE by acute sibutramine administration. **A:** Dose-response curves to methoxamine in the ventral prostate before (open symbols) and after (closed symbols) intravenous sibutramine 5 mg.kg^−1^ administration (n = 4 rats). **B:** Dose-response curves to NE in the ventral prostate before (open symbols) and after (closed symbols) intravenous sibutramine 5 mg.kg^−1^ administration (n = 5 rats). **A–B:** The data are expressed as the mean±SEM. * p<0.05 versus tension developed by the same NE dose before sibutramine administration (Student's t- test).

Cumulative intravenous administration of methoxanime induced dose-dependent tension in the ventral prostate of rats with high potency (pED_50_ = 4.60±0.24). After administration of 5 mg/kg sibutramine, the methoxamine dose-response curve showed similar potency (pED_50_ = 4.97±0.48) (Figure 4A).

Cumulative intravenous administration of NE induced dose-dependent tension in the ventral prostate of rats (pED_50_ = 4.78±0.59). After administration of 5 mg/kg sibutramine the NE dose-response curve was displaced to the left by approximately 3-fold due to increase in the tension developed by NE 1 and 3 µg/kg (Figure 4B), but the value of the potency was spurious (not showed).

**Figure 5:**
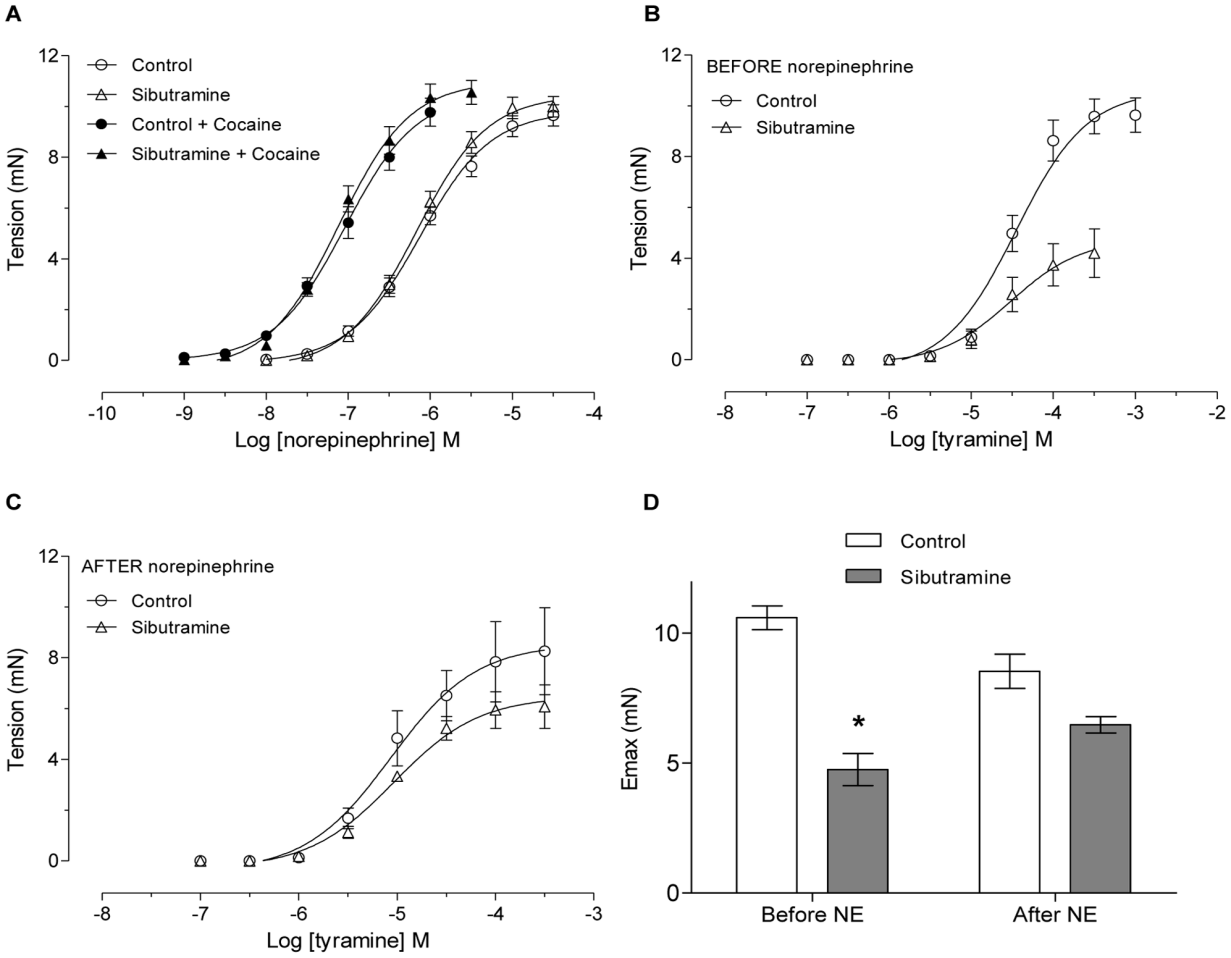
*In vivo* sibutramine administration decreases the tyramine-induced tension of epididymal cauda duct. **A:** Concentration-response curves to norepinephrine in control and sibutramine-treated rats in the absence (open symbols) and in the presence (closed symbols) of cocaine 6 µM. **B:** Concentration-response curves to tyramine in control and sibutramine-treated rats before concentration-response curve to exogenous norepinephrine. **C:** Concentration-response curves to tyramine in control and sibutramine-treated rats after concentration-response curve to exogenous norepinephrine. **A–C:** The data are expressed as mean±SEM. Values of pEC_50_ were not different between curves (Student's t- test). **D:** Maximal response (Emax) to tyramine before and after concentration-response curve to norepinephrine (NE) between control and sibutramine-treated rats. The data are expressed as the mean±SEM. *p<0.05 vs control (Student's t- test).

The potency of NE in the tension of the epididymal duct from rats treated with vehicle (control group, pEC_50_ = 6.128±0.059) was similar to the potency found in epididymal duct from rats treated with sibutramine *in vivo* (pEC_50_ = 6.170±0.057) (Figure 5A). Curves to NE obtained in the presence of 6 µM cocaine in tissues from rats from both groups were similar indicating that the neuronal NE reuptake system was not modified (Figura 5A), because cocaine is a potent norepinephrine reuptake inhibitor [30].

**Figure 6:**
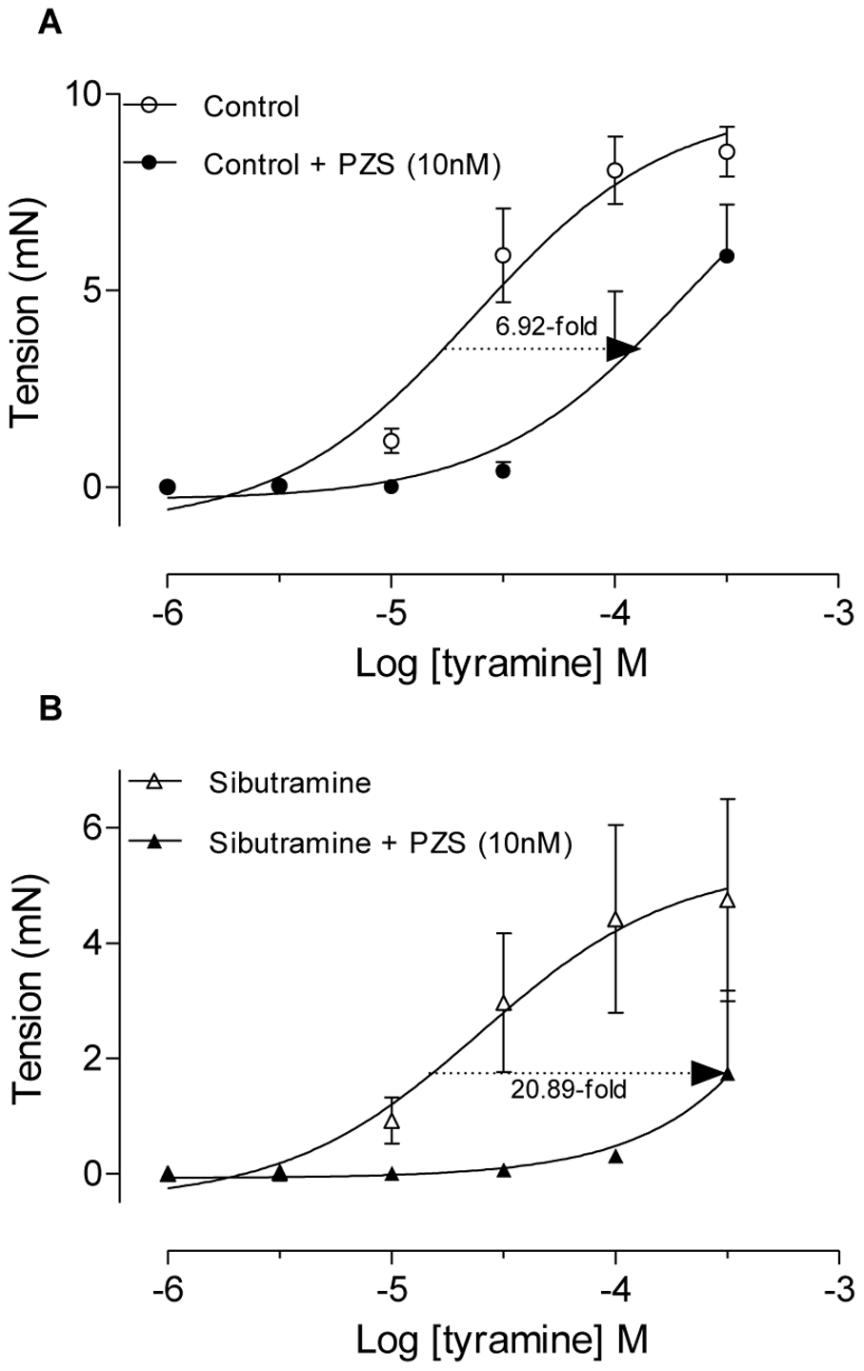
*In vivo* sibutramine administration increases the potency of prazosin-induced antagonism of epididymal tension to tyramine. Concentration-reponse curves to tyramine in the absence and presence of prazosin 10 nM (PZS) in the epididymal cauda from vehicle (**A**) and sibutramine (**B**) treated rats. Numbers under the arrows indicate the reduction in the potency of tyramine-induced tension of epididymal segments evaluated at 37% of maximal response of control curves of both groups. Data are mean ± SEM of tissues from 3 rats/group.

Tyramine, an amine that induces the release of NE stored in sympathetic varicosities, acting indirect sympathomimetic drug, presented similar potencies in the tension of the epididymal duct from rats treated with vehicle (control group, pEC_50_ = 4.520±0.107) and in ducts from sibutramine-treated rats (pEC_50_ = 4.577±0.282) (Figure 5B). However, the Emax for tyramine in epididymal ducts from rats treated with sibutramine was decreased (p<0.05, Figure 5B). Interestingly, the maximal response induced by tyramine in the epididymal cauda from sibutramine-treated rats, but not from control rats, was increased when a new concentration-response curve to tyramine (Figure 5C) was repeated 30 minutes after a concentration-response to NE (Figure S3); the difference between the maximal response to tyramine in the epididymal cauda from vehicle and sibutramine-treated rats was reduced (Figure 5D). These results suggest that the endogenous NE content in the epididymal cauda from sibutramine-treated rats is reduced. Figure 6 shows that prazosin, a selective α_1_-ARs competitive antagonist, was approximately 3-fold more potent in the epididymal cauda from sibutramine-treated rats than in the vehicle-treated rats, this can be seen by the reduction of the tension induced by tyramine, further suggesting a reduction in the endogenous NE content in the epididymal cauda from sibutramine-treated rats. The values of the pEC_50_ in the absence of the prazosin in the control (pEC_50_ = 4.627±0.160) and sibutramine-treated group (pEC_50_ = 4.603±0.415) were similar, but in the prazosin presence the values of pEC_50_ were not obtained, because did not attain the maximal contraction as revealed by the absence of the superior *plateau.*

## Discussion

Currently, there are some reports in the literature on the direct and indirect effects of sympathomimetic drugs on reproductive organs [12], [20], [34], [35], [36], [37], however there are no extensive studies on the mechanisms involved. In this study, we report pharmacological mechanisms by means sibutramine, a serotonin-NE reuptake inhibitor, promotes increased NE effects in the rat epididymal duct and ventral prostate and promotes NE depletion in the epididymis. These alterations were accompanied by reduced sperm quality and fertility.

Sibutramine inhibits the presynaptic reuptake of monoaminergic neurotransmitters in the central nervous system and this inhibition promotes appetite suppression and weight loss [18]. Thus, the reduction of body weight of the animals treated with sibutramine confirms the efficiency of this anorexigenic drug. However, it is known that increasing anorexigenic activity promotes increase in energy expenditure and induction of peripheral sympathomimetic effects such as blood pressure increase and other effects on different systems [18].

The determination of absolute and relative weights of organs provides important parameters for assessing the risk of toxicity on the male reproductive system [3], [38]. In the present study there was a reduction in the absolute and relative weights of the ventral prostate and epididymis as observed in previous work [20]. We also registered a significant reduction in the absolute weight of seminal vesicle. It is known that the autonomic nervous system plays an important role in growth, maturation and secretory function of the prostate, an androgen-dependent gland [39] and prostatic fluid release is modulated by the action of sympathetic fibers through stimulation of α1-ARs [8], [40]. As the testosterone levels of sibutramine-treated rats were not changed, the reduction of ventral prostate weight of sibutramine-treated animals may be due to the sympathomimetic effect of this drug [16], [41], [42]. Concentration-response curves with phenylephrine, an α1-adrenoceptor selective agonist, confirmed the presence of α1-adrenoceptors in the ventral prostate. As sibutramine can act inhibiting neuronal reuptake, concentration-response curves with methoxamine, a selective α_1_-adrenoceptor agonist that is not a substrate for the neuronal uptake system [29], were used to evaluate this effect. The results showed similar response in the absence or presence of sibutramine, unlike the concentration-response curves with NE that in sibutramine presence increased the ventral prostate tension developed. Thus, NE reuptake inhibition by sibutramine is the main mechanism that increases the tension of the ventral prostate to this neurotransmitter. As the seminal vesicle also receives a dense sympathetic innervations, it is very likely that the same mechanism was responsible for the decrease in the weight of this organ [12]. The reduction in epididymis weight may be due to the observed reduction in sperm reserves provoked by sperm transit time acceleration, as a result of the increased sensitivity of the epididymal duct as shown by the results of the pharmacological assays, as described below.

Sperm transit time through the epididymis plays an important role in post-testicular sperm maturation. The sympathetic innervation, mainly mediated by activation of α1-ARs, is associated with smooth muscle layer of the epididymal cauda, whose contractions mediate sperm passage through the epididymis [2], [5], [6]. Thus, drugs acting on the sympathetic system or directly on the tension developed of the epididymis, have an important impact on sperm transit time [23], [43], [44]. The present study corroborates previous works showing that an acceleration of sperm transit time in the epididymal cauda, as observed in sibutramine-treated rats, reduces sperm quality and fertility [3], [20], [24], [25].

In the present work, sibutramine *per se* tensed the epididymal duct and increased the potency of the NE in inducing tension of this duct. The increase in the mechanical activity of the distal epididymal cauda was abolished in the presence of nifedipine, a L-type calcium channel blocker [45]. As previous studies showed that sibutramine blocks voltage-dependent K^+^ channel [12], [20], [37], [46], [47] it is possible that the resulting membrane depolarization opens L-type calcium channels, allowing influx of extracellular calcium and contracting the distal epididymal cauda.

The increased potency of NE to induce epididymal duct contractions in presence of sibutramine can be explained by inhibition of neuronal reuptake [19]. This hypothesis is substantiated by our results showing that sibutramine increased the potency of NE, but not the potency of methoxamine, a selective α_1_-adrenoceptor agonist that is not a substrate for the neuronal uptake system [29]. Thus, NE reuptake inhibition by sibutramine is the main mechanism that increases the sensitivity of the epididymal cauda duct to this neurotransmitter.

Interestingly, the tension of epididymal duct isolated from sibutramine-treated rats to exogenous norepinephrine was not changed. This could be due to the *in vivo* drug metabolism, as the epididymal duct was isolated for the pharmacological reactivity 24 hours after the last sibutramine administration. In addition, the nutrient solution changes during the stabilization period *in vitro* may have removed all or part of the sibutramine in contact with the epididymal duct. Despite of unaltered epididymal duct tension to exogenous NE, through the pharmacological assays sub-chronic treatment with sibutramine reduced the epididymal cauda endogenous NE content, as shown by the reduced contraction of epididymal duct to tyramine. Tyramine, an indirect sympathomimetic drug, acts through the capture channels present in the cell membrane and is absorbed and accumulated in sympathetic varicosities, thus promoting the release of the norepinephrine stored in this varicosities [31], [32], [48]. This is further supported by the recovery of tyramine-induced maximal response after the addition of exogenous NE. In addition, prazosin, a competitive α1-AR antagonist, inhibited the tension induced by tyramine with higher potency in the sibutramine-treated group, possibly indicating a lower endogenous NE concentration. In fact, the potency of competitive antagonists against the effects induced by indirect agonists as tyramine are increased when the endogenous agonist pool (NE) is reduced [49].

Altogether, the pharmacological assays showed that sibutramine, in this experimental design, acts through independent mechanisms in the epididymal duct: reducing NE content in the epididymal sympathetic varicosities and increasing the NE potency in the smooth cells by inhibiting neuronal uptake. Furthermore, sibutramine promotes increased tension developed in the epididymal duct, mediated by calcium ions influx, as shown by the nifedipine assays. This finding was corroborated by a previous work [37], in which sibutramine pretreatment promoted an influx of calcium ions and increased the tension developed in the vas deferens.

In a previous work from our laboratory the rat fertility, assessed by natural mating, did not alter after sibutramine-treatment, despite a significant decrease in sperm reserves of the epididymal cauda [20]. Since rodents produce an excess of qualitatively normal sperm [26], it was necessary to use a technique to increase the sensitivity of the fertility test and obtain information on sperm quality. Moreover, this technique excludes the interference of factors such as altered sexual behavior pattern and reduction in ejaculated sperm numbers [3], [24], [25]. In the present work, fertility potential and the pre-implantation loss, complementary endpoints, were altered after sibutramine-treatment, suggesting that the acceleration of sperm transit time impaired sperm maturation in the epididymis, decreasing sperm quality and, consequently, fertility [3], [4]. Interestingly, although there was no statistically significant difference in sperm motility, this parameter showed a positive correlation with fertility results [20], [50].

In conclusion, our results confirm that adult rats subchronically exposed to sibutramine present significant reduction in epididymal sperm reserves, acceleration of sperm transit time, and NE potency increased in the epididymal duct. *In utero* artificial insemination revealed detrimental effects of sibutramine on fertility and sperm quality. The comprehensive pharmacological reactivity assays of epididymal cauda duct and ventral prostate, detailed described and validated, showed that a possible epididymal NE depletion exerted by sibutramine is associated with decreases in sperm transit time, quantity and quality, leading to reduced fertility. Our investigation reinforces the concerns about the possible impact on fertility of man taking sibutramine or other non-selective serotonin-norepinephrine reuptake inhibitors, especially when considering the lower reproductive efficiency of humans compared to other males. Additional investigations on the epididymal duct contractility and NE concentration, fertility after longer periods of sibutramine exposure and the possible recovery after treatment withdrawal are encouraged.

## Supporting Information

Figure S1 **Similar** ***in vivo*** **prostate tension to phenylephrine.** Dose-response curves to phenylephrine in the ventral prostate (n = 5 rats). The data are expressed as the mean±SEM. (Student's t- test).
(TIF)

Figure S2 **Effects of** ***in vitro*** **sibutramine 3 µM on basal tonus activity of distal epididymal duct.** **A:** Tension developed in 5 min in the absence of sibutramine. **B:** Tension developed in 5 min in the presence of sibutramine 3 µM. **C**: Tension developed in 5 min in the presence of sibutramine 3 µM and nifedipine 300 nM. **A–C:** The data are expressed as area under curve (AUC) of the 1 strip of the distal epididymal duct to show how the tension developed during 5 minutes is derived.
(TIF)

Figure S3 **The potency of NE** ***in vivo*** **sibutramine treatment was similar.** Concentration-response curves to norepinephrine in control and sibutramine-treated rats The data are expressed as the mean±SEM. Values of pEC_50_ were similar between NE curves (ANOVA, followed by Dunnett).
(TIF)

Table S1 **Serum hormonal levels.**
(DOC)
