# Supplementary figures and images for: Slimmer or Fertile? Pharmacological Mechanisms Involved in Reduced Sperm Quality and Fertility in Rats Exposed to the Anorexigen Sibutramine
Source: PLoS One. 2013 Jun 12;8(6):e66091. doi: 10.1371/journal.pone.0066091 (PMC3680400; doi:10.1371/journal.pone.0066091)

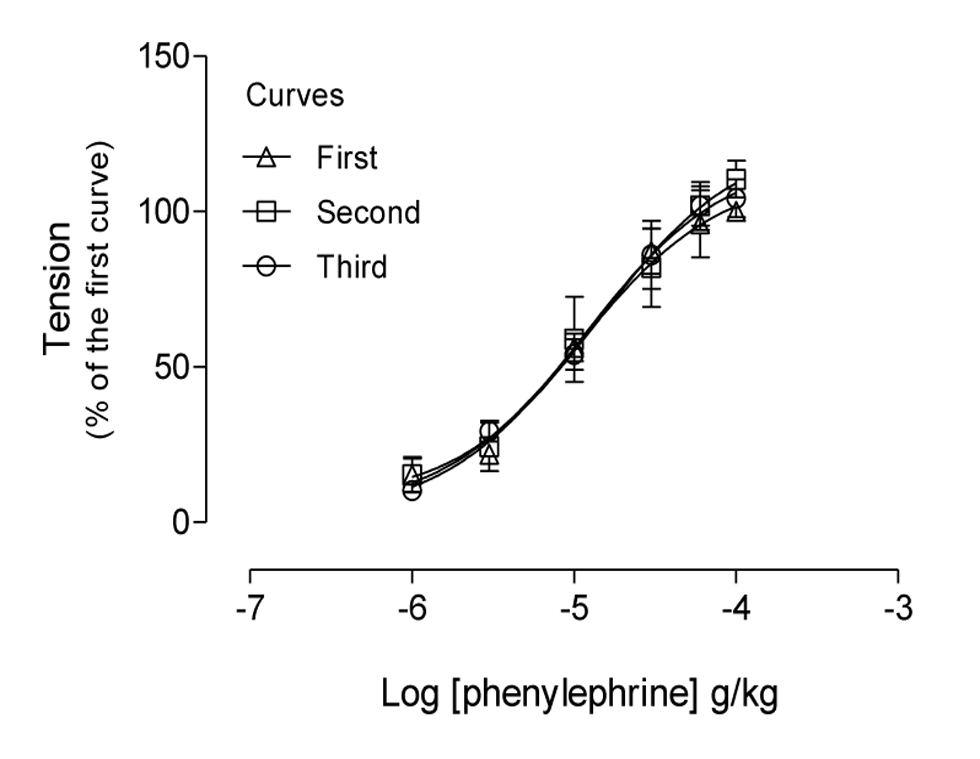

Supplement: Figure S1 — Similar in vivo prostate tension to phenylephrine. Dose-response curves to phenylephrine in the ventral prostate (n = 5 rats). The data are expressed as the mean±SEM. (Student's t- test). (TIF) [file pone.0066091.s001.tif]

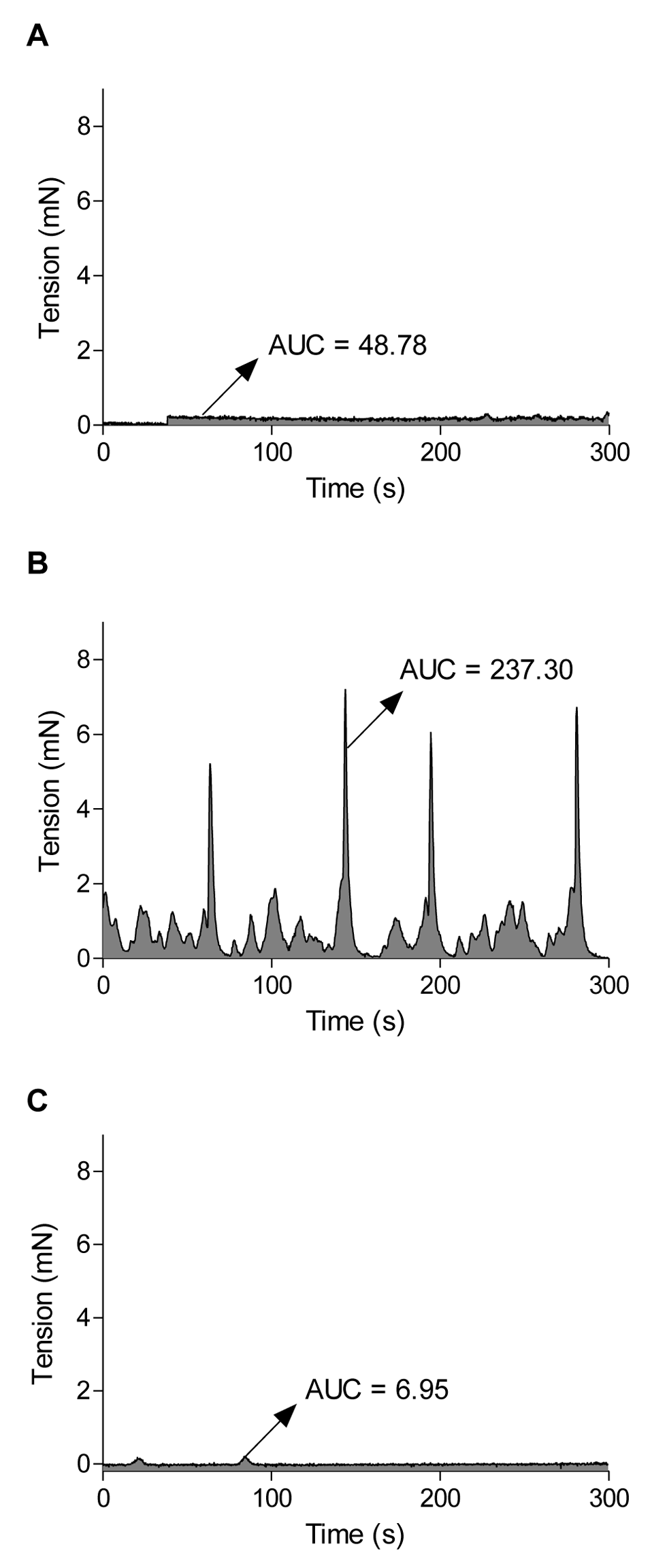

Supplement: Figure S2 — Effects of in vitro sibutramine 3 µM on basal tonus activity of distal epididymal duct. A: Tension developed in 5 min in the absence of sibutramine. B: Tension developed in 5 min in the presence of sibutramine 3 µM. C: Tension developed in 5 min in the presence of sibutramine 3 µM and nifedipine 300 nM. A–C: The data are expressed as area under curve (AUC) of the 1 strip of the distal epididymal duct to show how the tension developed during 5 minutes is derived. (TIF) [file pone.0066091.s002.tif]

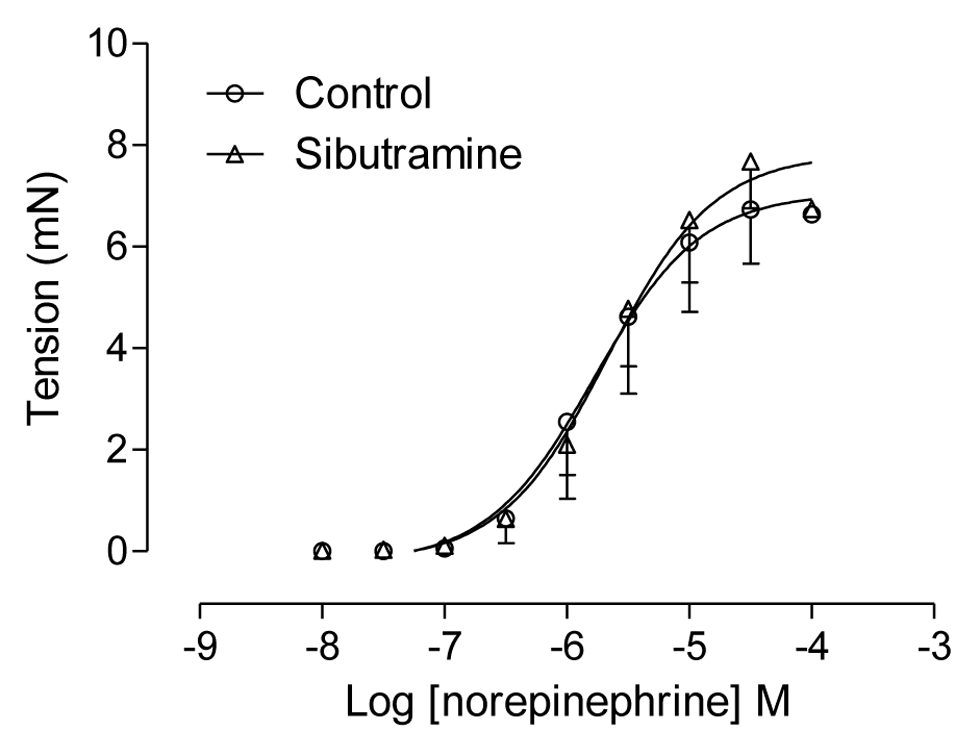

Supplement: Figure S3 — The potency of NE in vivo sibutramine treatment was similar. Concentration-response curves to norepinephrine in control and sibutramine-treated rats The data are expressed as the mean±SEM. Values of pEC50 were similar between NE curves (ANOVA, followed by Dunnett). (TIF) [file pone.0066091.s003.tif]
