# Supplementary material for: Slimmer or Fertile? Pharmacological Mechanisms Involved in Reduced Sperm Quality and Fertility in Rats Exposed to the Anorexigen Sibutramine
Source: PLoS One. 2013 Jun 12;8(6):e66091. doi: 10.1371/journal.pone.0066091 (PMC3680400; doi:10.1371/journal.pone.0066091)
Supplement: Table S1 — Serum hormonal levels. (DOC) [file pone.0066091.s004.doc]

**Table S1. Serum hormonal levels.**

| **Parameters** | **Control (n=8)** | **Sibutramine (n=8)** |
| --- | --- | --- |
| FSH (ng/mL) | 4.35 + 0.28 | 4.33 + 0.23 |
| LH (ng/mL) | 2.49 + 0.64 | 2.32 + 0.21 |
| Testosterone (ng/mL) | 3.55 + 0.56 | 3.37 + 0.40 |

Values expressed as mean + SEM. (Student's t- test).
